# Submission of mandatory respiratory health examinations among US coal miners participating in the Coal Workers’ Health Surveillance Program

**DOI:** 10.1136/oemed-2022-108644

**Published:** 2023-04-25

**Authors:** Noemi B Hall, Laura Reynolds, David J Blackley, A Scott Laney

**Affiliations:** Respiratory Health Division, National Institute for Occupational Safety and Health, Morgantown, West Virginia, USA

## Abstract

**Background:**

Mandatory examination requirements for US coal miners newly entering the workforce have been in place since the 1969 Coal Act mandated chest radiographs and were updated to include spirometry with promulgation of the 2014 Mine Safety and Health Administration Dust Rule. Compliance with the mandatory respiratory screening series is described using data from the National Institute for Occupational Safety and Health Coal Workers’ Health Surveillance Program (CWHSP).

**Methods:**

Among all radiographic and spirometry submissions to the CWHSP during 30 June 1971–15 March 2022, new underground coal miners who began work in the industry after 30 June 1971, and new underground, surface miners and contractors who began work after new regulations were implemented 1 August 2014, were identified and included in analysis.

**Results:**

Of the 115 093 unique miners who participated in the CWHSP and whose estimated entry into mining occurred during 30 June 1971–15 March 2019, 50 487 (43.9%) received their initial mandatory radiograph, and 15 452 (13.4%) submitted their initial and 3-year mandatory radiographs. Since new regulations were implemented, compliance with initial radiographs appeared to improve (80%) but compliance with 3-year radiographs remained low (11.6%). Compliance with spirometry testing was also low for initial (17.1%) and follow-up screenings (2.7%).

**Conclusions:**

The majority of new coal miners eligible for health surveillance did not receive a baseline radiograph or spirometry test through the CWHSP even though coal mine operators are required by law to provide these. Ensuring coal miners’ regular participation in health surveillance from early in their careers is an important way to monitor and protect their respiratory health.

## INTRODUCTION

In 1969, Congress passed the Federal Coal Mine Health and Safety Act (Coal Act) which established a range of measures to prevent lung disease in coal miners including periodic radiographic screening of underground coal miners throughout their working careers to identify coal workers’ pneumoconiosis. One provision of the law pertaining to radiographic surveillance of underground coal miners is that ‘Each worker who begins work in a coal mine for the first time shall be given, as soon as possible after commencement of his employment, and again 3 years later if he is still engaged in coal mining, a chest roentgenogram’.^1^ The Coal Act was superseded by the Federal Mine Safety and Health Act of 1977 (Mine Act) which enhanced miner safety and health standards and created the Mine Safety and Health Administration (MSHA), transferring enforcement responsibilities from the Department of the Interior to the Department of Labor.^2^ The mandatory radiographic screening of new miners remained unchanged in the Mine Act and was implemented in subsequent rulemaking by the Department of Labor (MSHA) and Health and Human Services (National Institute for Occupational Safety and Health (NIOSH)).^3 4^ The text of the rules have undergone slight revision over time, however, the intent of mandated radiographs on commencement of employment and within 3 years thereafter has remained unchanged for the subsequent 53 years.

In 2014, MSHA published a final rule which extended medical screening to include surface and contract coal miners (those coal workers who are not directly employed by the mine itself, but are employed through a contracting company) and expanded medical surveillance requirements to add spirometry testing and a respiratory health questionnaire to the periodic chest radiographic exams.^4^ NIOSH subsequently updated its standards to incorporate these expanded screening requirements.^3^ The 2014 MSHA dust rule specifies that for each miner who begins work at a coal mine for the first time, the operator shall provide chest radiograph and spirometry examinations no later than 30 days after beginning employment and follow-up examinations no later than 3 years after the initial examination.

Since implementation of the 1969 Coal Act, a chest radiograph should have been obtained for every underground coal miner new to the industry shortly after employment and within 3 years thereafter. From the implementation date specified in NIOSH’s 2014 Interim Final Rule, which implemented surveillance required in the 2014 MSHA Dust Rule, this mandate also applies to surface and contract miners, and requires spirometry for new miners at the same time intervals. In this study, we assess compliance with the mandated respiratory screening required for miners new to the industry, highlight obstacles that may prevent complete adherence to the mandate and discuss potential solutions for improvement.

## METHODS

### Subjects

NIOSH (and the US Public Health Service before it) has administered the Coal Workers’ Health Surveillance Program (CWHSP) to conduct surveillance for occupational respiratory disease among working US coal miners since 1970.^5–7^ The CWHSP provides radiographic screening for working miners throughout their careers at no cost to themselves. These screenings can be acquired either at NIOSH-approved facilities, as described in each operator’s mine plan, or through the CWHSP mobile examination unit, which has been in operation since 2005.^8^ As stated in the 1969 Coal Act, new miners who began work in the underground coal mining industry after 30 June 1971, must submit their mandatory initial screening to NIOSH within 6 months of employment. Following the 2014 MSHA and NIOSH rules, the guidelines and timeline shifted, so that new miners who entered the coal mining industry on or after 1 August 2014, including underground, surface and contract miners must submit their mandatory initial screening to NIOSH within 30 days of employment. For all miners who entered the coal mining industry after 30 June 1971, a second radiographic screening is also mandatory, to be completed within 3 years following the date of the initial radiograph submitted to the CWHSP.

The 2014 MSHA Dust Rule also introduced mandatory spirometry testing for new underground, surface and contract miners to be submitted within 30 days of employment and a second spirometry test within 3 years following the date of the initial spirometry test submitted to the CWHSP. Following 2016 amendments to 42 Code of Federal Regulations (CFR) part 37, specifications for medical examinations of coal miners, the implementation date for mandatory spirometry screenings for new miners was after 30 November 2018.^3^

Miners new to the industry are not reported directly to NIOSH. They are only known to NIOSH once their initial radiograph or spirometry test is submitted to the CWHSP and the accompanying documentation identifies them as having zero years of tenure working as a coal miner. NIOSH B Readers, physicians who are trained to classify radiographs according to the International Labour Office standards,^9^ classify the radiograph and NIOSH interprets spirometry results. The miner is then notified of the results by a letter mailed directly to their home mailing address on file. Two years and 6 months after the date of the initial radiographic or spirometry screening, a reminder notice is mailed to the operator and to the miner that the second mandatory radiograph and spirometry test must be obtained and transmitted to NIOSH.

### Analysis

All data used for analysis come from the CWHSP, which includes radiographic and spirometry screenings. All radiographs submitted to the CWHSP during 30 June 1971–15 March 2022 were included in this analysis. If a miner has never submitted a respiratory health screening to the CWHSP, then they are necessarily excluded from this analysis. The beginning date for this time period is the effective date for compliance with the submission of mandatory radiographs for new miners: 18 months after enactment of the Coal Act on 30 December 1969. All spirometry testing results submitted to the CWHSP during 1 December 2018–15 March 2022 were also included in analysis. The beginning date for this time period is the effective date for compliance with the submission of mandatory spirometry test results for new miners. The most recent date included for the estimated start date of a new miner was 15 March 2019, to allow the full 3 years of follow-up by 15 March 2022.

Four different time frames were defined for the estimated starting date of miners: 30 June 1971–15 March 2019; 30 June 1971–31 July 2014; 1 August 2014–15 March 2019; and 1 December 2018–15 March 2019. The first time frame encompasses the entire set of miners who were eligible and participated in the CWHSP following the 1969 Coal Act. The second time frame includes only those underground coal miners who started working in the industry after the enactment of the 1969 Coal Act and before the 2014 MSHA Dust Rule was enacted on 1 August 2014. The third time frame includes only those underground, surface and contract coal miners who started working in the industry after the enactment of the 2014 MSHA Dust Rule. The fourth time frame includes only those underground, surface and contract coal miners who started working in the industry after the implementation of mandatory spirometry screenings.

A radiograph or spirometry test submitted was defined as a new miner submission if it was the first submitted for a given miner and the tenure reported for the miner was 0 years. The miner’s estimated date of entry into the industry was then recorded as the date the radiograph had been taken. If the miner’s tenure was missing from their initial radiographic submission, but a second radiograph was submitted with reported tenure available, then the miner’s estimated date of entry into the industry was calculated based on the date the second radiograph was submitted minus the number of years of reported tenure. Otherwise, radiographs submitted with missing tenure information were removed from analysis.

All data analyses were conducted by using SAS V.9.4.

## RESULTS

Among the 115 093 unique miners who participated in the CWHSP during 30 June 1971–15 March 2022 and whose estimated start date in the industry was between 30 June 1971 and 15 March 2019, 50 487 (43.9%) submitted their mandatory new miner radiographic screening (table 1). Among these miners who submitted an initial new miner radiograph, 15 452 (30.6%) also submitted a follow-up radiograph within 3 years of their initial submission. Overall, 13.4% of the eligible miners participating in the CWHSP submitted the complete mandatory radiograph series.

Among the 102 534 underground coal miners who participated in the CWHSP and entered the industry during the time frame covered by the 1969 Coal Act, 30 June 1971–31 July 2014, 13.9% of eligible miners submitted their mandatory radiograph submission series.

Among the 12 554 miners who participated in the CWHSP following the enactment of the 2014 MSHA Dust Rule and entered the industry during 1 August 2014–15 March 2019, 10 091 (80%) submitted their new miner radiographic screening and 1174 (11.6%) of those also submitted their radiographic follow-up, which means that 9.3% of eligible miners in this time frame submitted the mandatory radiograph submission series (table 2).

Among the 12 554 new miners who participated following the enactment of the 2014 MSHA Dust Rule, submission of the initial new miner radiograph was highest among contract miners (85.8%), compared with 77.6% of surface miners and 70.7% of underground miners.

Among the 847 new miners who participated following the first 4 months of implementation of spirometry testing during 1 December 2018–15 March 2019, spirometry was submitted with 125 (17.1%) of the initial 731 new miner radiographs. Among the miners who submitted their radiographic follow-up within 3 years, 2 (2.7%) miners also submitted spirometry.

## DISCUSSION

Since enactment of the 1969 Coal Act, most underground coal miners new to the industry did not receive a baseline radiograph despite it being required by law. These data suggest frequent non-compliance with the mandatory respiratory health screenings. It is likely that multiple factors are responsible for miners not receiving their mandatory baseline chest radiograph and include a lack of systematic identification of new miners in the industry, lack of knowledge of the requirements by new miners and operators, lack of enforcement by MSHA inspectors, and, most recently, the COVID-19 pandemic.

Similarly, following the implementation of spirometry testing for new miners in 2018, only a small portion of miners new to the industry received their baseline spirometry testing. While multiple factors may also be responsible for miners not receiving their mandatory baseline spirometry screening, the most important factor may be access to NIOSH-approved health facilities offering spirometry testing. Compared with the regulation for radiographic screening for new miners, which has been in place for over 50 years, spirometry testing has been mandatory for new miners only since implementation in 2018. The infrastructure needed to ensure miners have access to spirometry testing, just as they do with radiographs, continues to expand to meet the needs of the workforce and comply with regulations. There are 42 spirometry clinics (31 of which also offer radiographic screenings) which have been certified by NIOSH as of June 2022 in addition to spirometry testing available through the CWHSP mobile unit, an increase from the initial 30 health facilities that were certified by NIOSH to perform spirometry testing when it was first implemented by CWHSP in 2018. As of June 2022, of the 926 mines with active mine plans, 626 (67%) include a spirometry clinic within 50 miles of the mine site.

One difficulty in ensuring that miners receive entry medical examinations is that there is no formal tracking of new miners into the industry. Operators are required to file quarterly reports to MSHA regarding the number of currently employed miners at each mine, however, this does not distinguish between new miners, currently employed miners and new hires with experience. Though there are requirements for new miner safety training and medical examinations, the operators maintain these records for review rather than actively reporting to MSHA or NIOSH when new miners enter the industry. Therefore, it is not possible to clearly assess accurate rates of compliance with mandatory medical examinations because the denominator (total number of new miners) is unknown. In the last 20 years, voluntary participation in the CWHSP has been between 30% and 45%.^10^ If a coal miner decides to opt out of respiratory health screenings throughout their career, NIOSH does not have the ability to readily identify them. Though mine plans submitted to NIOSH do include rosters of current employees, the limited information available is not sufficient to identify unique miners, nor are new miners identified as such. The timeliness of mine plan submission to NIOSH and quarterly reports to MSHA are also not useful for ensuring miners participate in respiratory health screenings within the first 30 days of employment, as the regulations require.

Of the miners who did have an initial examination performed on entering the industry, less than one-third returned for the mandated second radiograph within 3 years. There are a number of possible reasons for this observation. First, the miner may have refused the radiograph for a variety of reasons, including fear that participation in surveillance might adversely affect their employment.^11 12^ Second, the miner may have been unaware of the requirement. For example, they might have moved from their previous residence and did not receive notification of the requirement. Third, the employer did not inform or remind the miner of the need to participate in this mandatory follow-up screening. This may be in part be due to infrequent enforcement of this regulation by MSHA inspectors, as described later in the discussion, and a subsequent lack of penalties for the mine operator. Fourth, the miner may have changed employment to a different mine. Finally, the miner may have left the industry all together. Because employment entry and exit are not systematically tracked it is unclear which of these factors is most responsible for the low rate of participation with the second mandated radiograph for coal miners.

The justification MSHA used in the 2014 Dust Rule for mandatory early career respiratory examinations was the necessity to establish an accurate baseline of each miner’s health.^4^ Although radiographic findings of pneumoconiosis in new, relatively unexposed miners are not expected, baseline chest radiographs can document radiographic appearance at entry into the profession, providing a basis for later comparisons. Early screening is important with regard to the mandatory spirometry component of the rule, since previous research has shown that declines in lung function due to dust exposure is non-linear and can manifest with more accelerated decline early in a miner’s career.^13 14^ MSHA notes that ‘there are some individuals who respond adversely to respirable coal dust exposure relatively quickly, and it is important to identify those individuals early’.^4^ To identify these early changes, it is useful to compare spirometry test results for an individual serially to their own baseline, since an individual can lose significant lung function before their tests are identified as abnormal in comparison to reference values based on the general population. This is especially important for a younger healthy working population, which may enter into mining with better baseline lung function than the general population.^15^

An additional justification for mandatory respiratory health screenings for new miners is that these early screenings familiarise new miners with the CWHSP and introduce them to the available benefit of voluntary regular screenings throughout their career. Though this screening is provided at no cost to the miner themselves, there is no regulatory requirement to cover the cost of transportation to the screening location or to account for time spent away from work to obtain the screening. Early participation may encourage miners to continue participating in CWHSP, which would then increase the likelihood of identifying disease at its earliest stages. If MSHA inspectors increase the enforcement frequency of this regulation, mine operators may also encourage participation in the CWHSP, not just for the initial mandatory respiratory health screenings, but throughout the miner’s career. Overall increased participation in CWHSP may then lead to overall increased utilisation of rights for coal miners with evidence of pneumoconiosis to be transferred to jobs with low dust exposure under the part 90 programme, which is intended to protect miners’ respiratory health and prevent progression to severe disease.

Though we are currently unable to assess the entire magnitude of the problem, the findings from this analysis demonstrate that non-compliance with mandatory respiratory health examinations for coal miners has historically been more common than compliance. We are unable to fully measure the impact of the 2014 MSHA Dust Rule on level of compliance due to the restricted 8-year time period (2014–2022), as some miners may wait many years before undergoing their first respiratory health screening.^16 17^ We are unable to calculate how many additional miners began working in the mining industry in this time period and have not yet interacted with the CWHSP system. However, this subset of data appears to show that compliance with mandatory radiographic examinations increased after implementation of the 2014 Dust Rule, with 80% of those identified as new miners submitting an initial radiograph (table 1). This apparent increase is driven largely by inclusion of surface miners and contractors who appear to have higher rates of participation than underground miners (77.6% and 85.8% of new surface miners and contractors, respectively, compared with 70.7% of new underground miners). However, at least 20% overall failed to submit an initial radiograph. Again, it is unknown how many new miners who did not provide a radiograph during this period may decide to participate in the CWHSP later in their career, and thus have not yet been identified as non-compliant with their mandatory new miner screenings.

Non-compliance with mandatory medical examinations is a citable offence for coal mine operators, highlighted in section 203 of the MSHA enforcement programme policy manual.^18^ However, only 18 instances have been identified in which a citation was levied based on non-compliance with mandated medical examinations (MSHA, personal communication, 27 July 2022). An internal review found no instance in which NIOSH referred a case to MSHA for review. Thus, to our knowledge, the mandate for required medical examinations specified in MSHA and NIOSH regulations has rarely been enforced.

It should be acknowledged that the COVID-19 global pandemic disrupted routine healthcare and occupational health screening, particularly spirometry, which is an aerosol-generating procedure with the potential to increase virus transmission in healthcare settings.^19^ This disruption in service began early in 2020 and the effects continue to this day with ‘normal’ services still yet to be fully restored. The NIOSH mobile examination unit has not been active since March 2020 and many of the fixed medical facilities participating in CWHSP were limited in their ability to provide surveillance services. Because of concerns about risk of COVID-19 transmission, NIOSH-approved spirometry facilities were advised to suspend testing for the CWHSP from March 2020 to July 2021. As a result, the number of respiratory health screenings for new miners conducted or received by NIOSH during this period declined precipitously compared with previous years. NIOSH received 2366 radiographs from new miners in the 2.5 years since 20 January 2020 (the date of the first laboratory-confirmed case of COVID-19 in the USA). The average 946 radiographs received annually during this time frame is in contrast to the number of new miner radiographs received in 2018 (2998) and 2019 (2386). The low numbers of radiographs received during the pandemic (44% less than the previous 2-year period) may reflect a lack of hiring in the subsequent 2 years but more likely reflects the lack of available screening opportunity for new miners. There is likely a large pool of new miners who were unable or unwilling to participate during the pandemic that are not accounted for in this analysis. Because there is no systematic accounting of new miners to the industry, it is currently unclear how many new miner screenings were not performed as a result of COVID-19 during this period.

The focus of this study is compliance with federal regulations regarding mandatory respiratory health examinations in the USA. However, regulations regarding respiratory health surveillance and mandatory screening for disease among coal workers do vary by country.^20–23^ While this limits the application of these findings to that of other top coal producing countries, regulations pertaining to medical surveillance generally rely on a functioning and robust system where federal regulation and enforcement play a vital role in industry compliance, regardless of location.

There are steps that operators, miners and government can take to improve and ensure compliance with new miner examinations in the USA. First, operators should be aware of the requirements which are clearly included in regulations and ensure all new miners are receiving the mandatory health examinations. For each employee who does submit an initial radiograph, NIOSH conducts outreach to the responsible mine operator, sending a letter to remind them of the mandatory 3-year follow-up respiratory health screening for the employee. Miners should be informed during initial safety training that this is a requirement and they should undergo this confidential health screening. The hiring of new miners could potentially be directly reported to either NIOSH or MSHA, as these agencies could play important roles in communicating with operators and miners about mandatory medical requirements.

NIOSH is committed to reducing barriers to participation by improving access to approved medical facilities for miners to receive medical examinations, including through use of the NIOSH mobile examination unit. It would be beneficial to have systems in place to identify new miners within the first 30 days of employment for targeted outreach to improve participation. For example, operators could document the completion of mandatory health examinations as they do with mandatory safety training. If this were done, then when inspecting the completion of safety training certificates MSHA inspectors could simultaneously confirm that new miners have records of completion of mandatory health examinations and take steps to enhance compliance. Tracking the respiratory health of miners from the very outset of their careers in the industry should be a priority.

## Figures and Tables

**Table 1:** Submission of mandatory new miner radiographic screening series among Coal Workers’ Health Surveillance Program (CWHSP) participants, 30 June 1971–15 March 2019

	Participation during 30 June 1971–15 March 2022, with estimated start during 30 June 1971–15 March 2019	Participation during 30 June 1971–15 March 2022, with estimated start during 30 June 1971–31 July 2014	Participation during 1 August 2014–15 March 2022, with estimated start during 1 August 2014–15 March 2019
Total miners participating in CWHSP	115 093	102 534	12 554
Miners with a new miner screening submitted*	50 487 (43.9%)	40 391 (39.4%)	10 091 (80%)
Miners with follow-up within 3 years of new miner screening†	15 452 (30.6%)	14 278 (35.3%)	1174 (11.6%)

* Percentage provided out of total participating miners in the given time frame.

† Percentage provided out of miners who submitted new miner screenings in the given time frame.

**Table 2:** Submission of mandatory new miner radiographic and spirometry screening among Coal Workers’ Health Surveillance Program (CWHSP) participants, 1 August 2014–15 March 2019

	N (%)	Underground miners	Surface miners	Contract miners
Total miners participating in CWHSP between 1 August 2014 and 15 March 2022, with estimated start date between 1 August 2014 and 15 March 2019	12 554	2232	4242	6080
Miners with a new miner radiographic screening submitted by 15 March 2019*	10 091 (80%)	1579 (70.7%)	3293 (77.6%)	5219 (85.8%)
Miners with radiographic follow-up within 3 years	1174 (11.6%)	346 (21.9%)	575 (17.4%)	253 (4.8%)
Total miners participating in CWHSP between 1 December 2018 and 15 March 2022, with estimated start date between 1 December 2018 and 15 March 2019	847	238	240	369
Miners with a new miner radiographic screening submitted by 15 March 2019*	731 (86.3%)	219 (92.0%)	197 (82.1%)	315 (85.3%)
With spirometry screening‡	125 (17.1%)	5 (2.3%)	32 (16.2%)	88 (27.9%)
Miners with radiographic follow-up† within 3 years	75 (10.2%)	20 (9.1%)	44 (22.3%)	11 (3.5%)
With spirometry screening‡	2 (2.7%)	1 (5%)	1 (2.3%)	0

* Percentage provided out of total participating miners in the given time frame.

† Percentage provided out of miners who submitted new miner screenings in the given time frame.

‡ Percentage provided out of miners who submitted radiographic screenings in the given time frame.

## Data Availability

Data are available on reasonable request. cwhsp@cdc.gov for further inquiry.

## WHAT IS ALREADY KNOWN ON THIS TOPIC

- Coal miners in the USA are required to participate in respiratory health screening at the outset of their mining careers, and again within 3 years of the initial screening. Early identification of pneumoconiosis, commonly known as black lung disease, and subsequent intervention is needed to prevent progression of disease.

## WHAT THIS STUDY ADDS

- The majority of new coal miners do not participate in respiratory health screenings, despite coal mine operators being federally mandated to require these screenings.

## HOW THIS STUDY MIGHT AFFECT RESEARCH, PRACTICE OR POLICY

- This study shows that the current system is not sufficient to ensure miners new to the industry are receiving and subsequently submitting their mandatory respiratory health screenings. To improve participation, systems may need to be put in place to identify these new miners within the first 30 days of employment for targeted outreach and to emphasise to mine operators the value of these screenings in protecting the health of the workforce.
